# Role of long non-coding RNAs in disease progression of early stage unmutated chronic lymphocytic leukemia

**DOI:** 10.18632/oncotarget.26538

**Published:** 2019-01-01

**Authors:** Renee C. Tschumper, Tait D. Shanafelt, Neil E. Kay, Diane F. Jelinek

**Affiliations:** ^1^ Department of Immunology, Mayo Clinic, Rochester, MN, USA; ^2^ Department of Immunology, Mayo Clinic, Scottsdale, AZ, USA; ^3^ Department of Hematology/Oncology, Stanford University, Stanford, CA, USA; ^4^ Department of Internal Medicine, Division of Hematology, Mayo Clinic, Rochester, MN, USA

**Keywords:** long non-coding RNA, chronic lymphocytic leukemia, unmutated immunoglobulin heavy chain variable region, progression, early Rai stage

## Abstract

Predicting disease progression in chronic lymphocytic leukemia (CLL) remains challenging particularly in patients with Rai Stage 0/I disease that have an unmutated immunoglobulin heavy chain variable region (UM IGHV). Even though patients with UM IGHV have a poor prognosis and generally require earlier treatment, not all UM IGHV patients experience more rapid disease progression with some remaining treatment free for many years. This observation suggests biologic characteristics other than known prognostic factors influence disease progression. Alterations in long non-coding RNA (lncRNA) expression levels have been implicated in diagnosis and prognosis of various cancers, however, their role in disease progression of early Rai stage UM CLL is unknown. Here we use microarray analysis to compare lncRNA and mRNA profiles of Rai 0/I UM IGHV patients who progressed in <2 years relative to patients who had not progressed for >5 years. Over 1,300 lncRNAs and 940 mRNAs were differentially expressed (fold change ≥ 2.0; p-value ≤ 0.05). Of interest, the differentially expressed lncRNAs T204050, NR_002947, and uc.436+, have known associated genes that have been linked to CLL. Thus, our study reveals differentially expressed lncRNAs in progressive early stage CLL requiring therapy versus indolent early Rai stage UM CLL. These lncRNAs have the potential to impact relevant biological processes and pathways that influence clinical outcome in CLL.

## INTRODUCTION

Chronic lymphocytic leukemia (CLL) remains a clinically heterogeneous disease despite recent advances in disease classification centered upon cellular and molecular markers. Prognostic models based on Rai stage, immunoglobulin heavy chain variable region mutation status (IGHV), cytogenetic abnormalities, gene mutations and expression of ZAP-70, CD38 and CD49d proteins have been developed and show efficacy in predicting disease outcome in some cases of CLL [1–10]. A recently developed CLL International Prognostic Index using age, clinical stage, *TP53* status, IGHV mutation status, and serum β2-microglobulin has been shown to better predict progression in early CLL [2, 11]. Despite these advances, heterogeneity in clinical behavior remains incompletely explained, particularly as it concerns the variable rate of progression that exists in early stage Rai (0/I) patients with an unmutated (UM) IGHV. Although UM IGHV status has been shown to be a negative prognostic factor [12, 13], some patients experience rapid progression and early death while others live for many years without disease progression. This disparity suggests that there are currently unidentified biologic factor(s) which contribute to CLL disease heterogeneity and progression within the Rai 0/I UM subset of CLL patients.

Gene expression profiling and genome-wide association studies have identified important genes and alleles that may play a pathobiological role in CLL [14–17]. However, these studies have focused on protein-coding genes and well-known annotated genes. Protein-coding genes account for only 2% of the human genome but more than 70% of the genome is transcribed into non-coding RNA [18]. Accumulating evidence demonstrates some functionality for non-coding RNAs. For example, small non-coding micro-RNAs have been shown to function as post-transcriptional regulators and play a role in differentiation and development in various diseases including CLL [19–25]. More recently, a number of studies have shown that the more complex long non-coding RNAs (lncRNAs) also play an important role in both cell development and cancer [26–30].

LncRNAs are transcribed RNA molecules greater than 200 nucleotides long and have been traditionally defined as having no open reading frame, although many lncRNAs associate with ribosomes and may encode short peptides [31, 32]. Studies of lncRNA expression in B cell development [33, 34] indicate a role for lncRNAs in the pathogenesis and progression of B cell malignancies [35, 36]. Indeed, there is evidence that lncRNAs can associate with clinical features in human acute and chronic leukemias allowing for discrimination of disease subtypes and clinical outcomes [37–45].

In this study, we used a microarray based approach to analyze the lncRNA and mRNA expression profiles of Rai 0/I UM CLL patients with progressive disease requiring therapy (PD; time to first treatment (TTT) ≤2 years) compared to those with more stable, indolent disease (ID; TTT ≥5 years). Our comprehensive profiling has allowed us to identify specific lncRNAs that are differentially expressed and may contribute to the clinical heterogeneity observed in this subset of CLL patients. Moreover, the data provide a crucial foundation for future studies investigating the utility of any of these lncRNAs to serve as biomarkers that may allow earlier prediction of disease progression.

## RESULTS

### LncRNA and mRNA expression profiles in early stage IGHV UM CLL patients with progressive versus indolent disease

**Figure 1:**
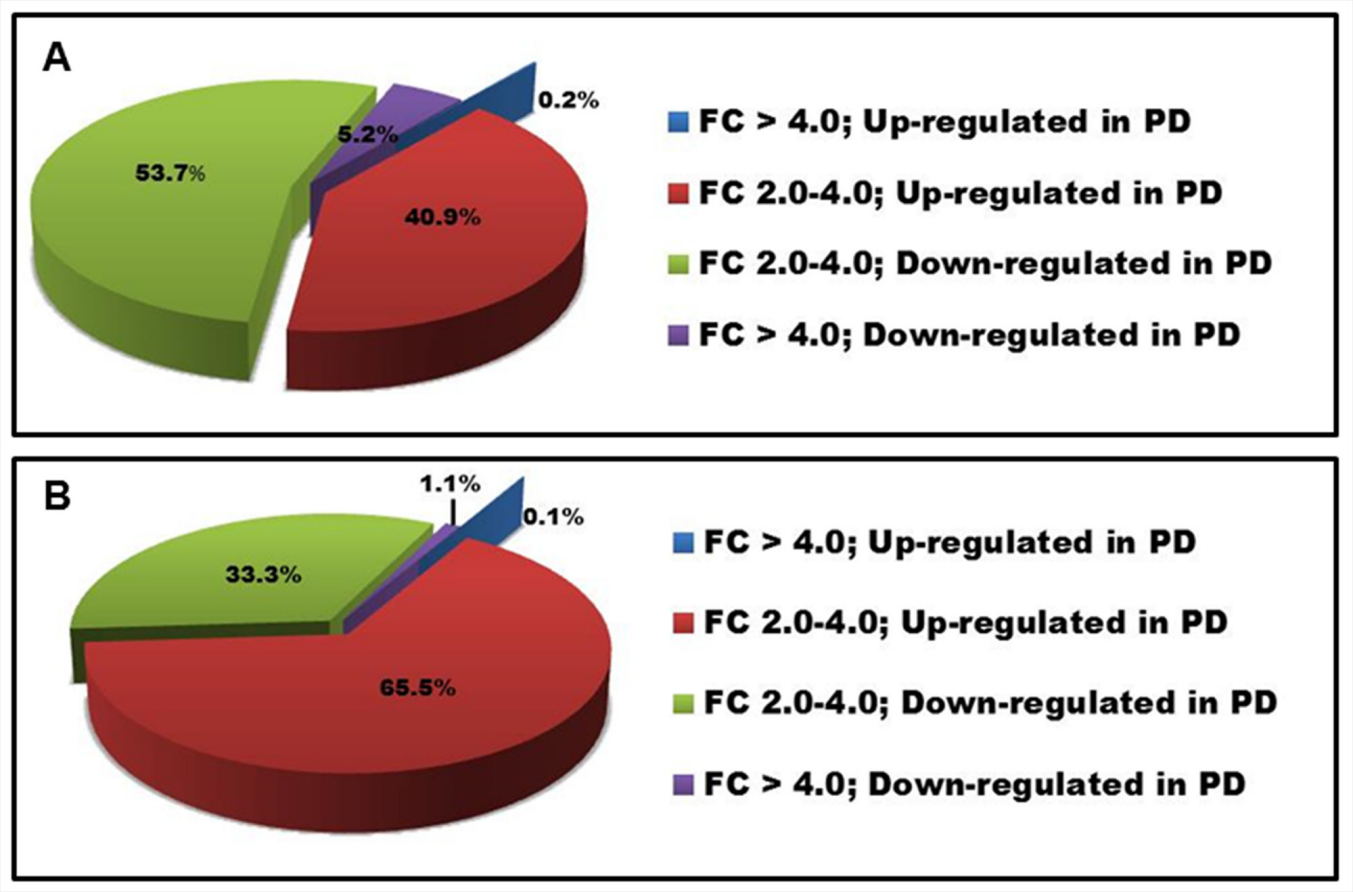
Distribution of differentially expressed lncRNAs and mRNAs in PD vs ID CLL FC in **(A)** lncRNA and **(B)** mRNA expression.

**Figure 2:**
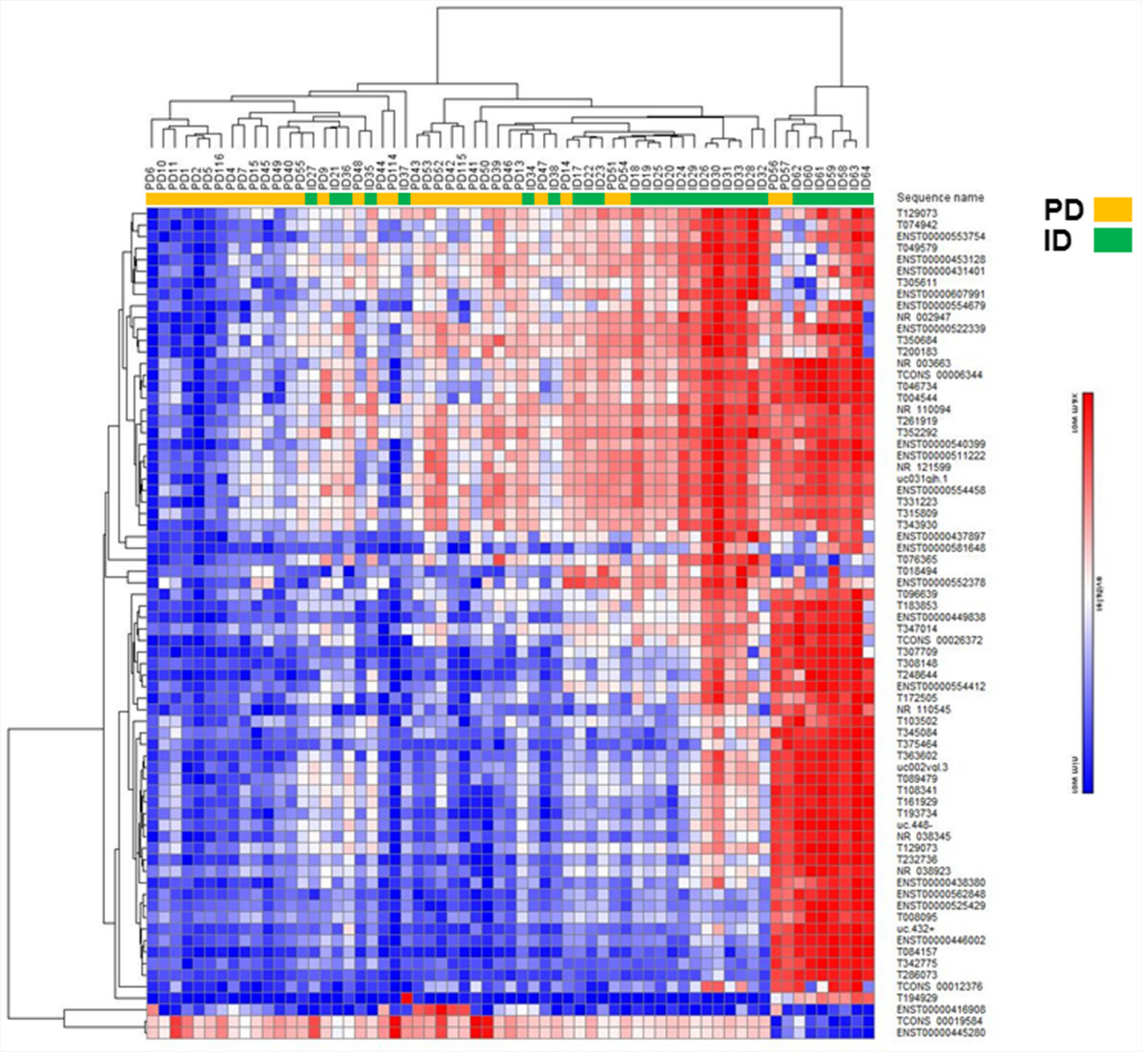
Hierarchical clustering analysis of differentially expressed lncRNAs in PD vs ID early stage CLL Heatmap of lncRNAs differentially expressed by FC ≥ 4.0.

**Figure 3:**
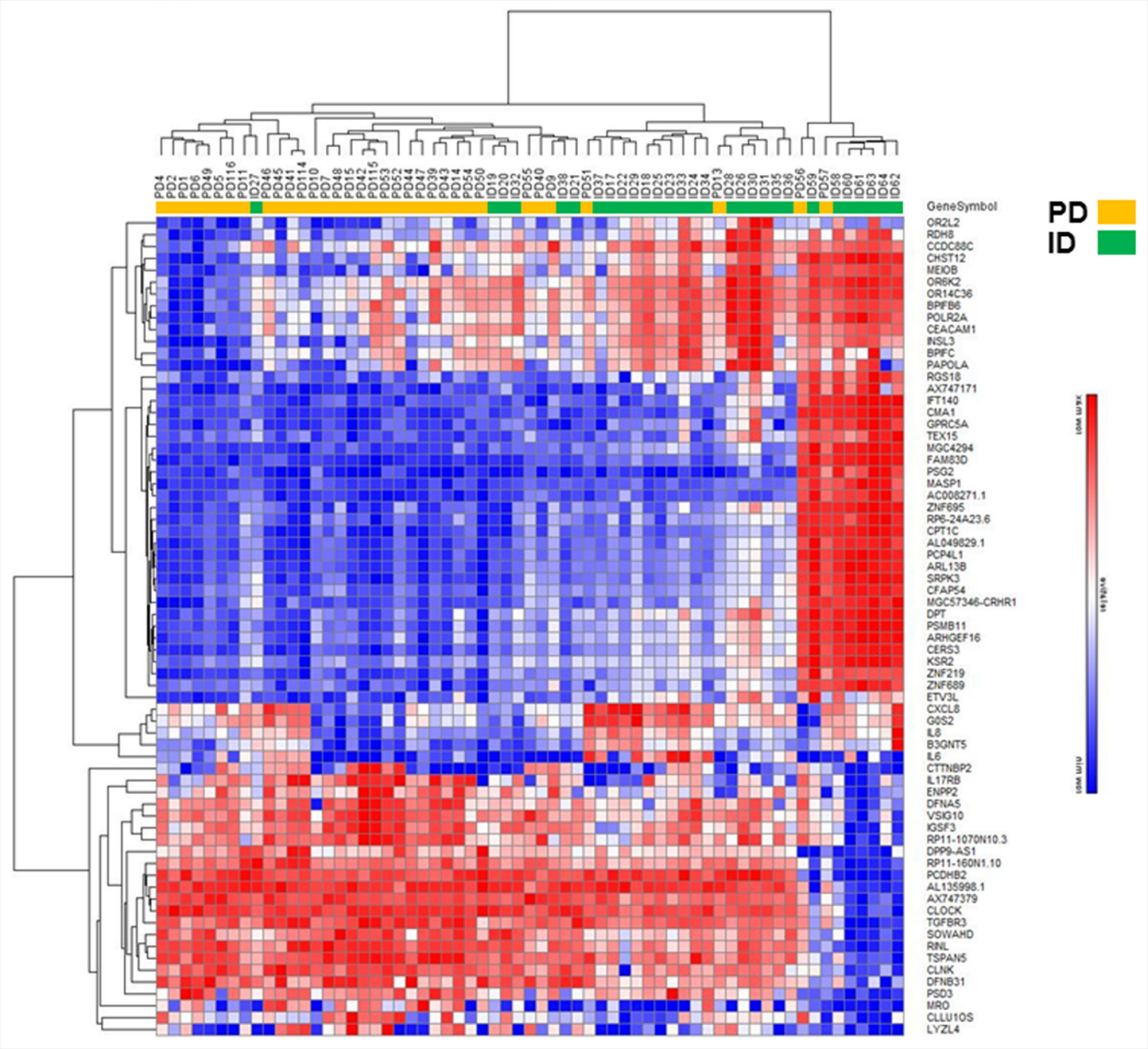
Hierarchical clustering analysis of differentially expressed mRNAs in PD vs ID early stage CLL Heatmap of mRNAs differentially expressed by FC ≥ 4.0.

LncRNA and mRNA expression profiling was performed on 34 PD and 29 ID Rai 0/I UM IGHV CLL samples using a microarray based approach (Table 1; Supplementary Table 1). Of the 40,173 lncRNAs on the Arraystar Human lncRNA Array v4.0, 1,330 lncRNAs (3.3%) were considered differentially expressed among PD compared to ID patients based on at least 12 Present or Marginal flags, a fold change (FC) ≥ 2.0 and a p-value ≤ 0.05 (as calculated by the t-test) after normalization. False discovery rates (FDRs) were adjusted from all the p-values by the Benjamini-Hochberg method for multiple testing corrections (data accessible at NCBI GEO database, accession GSE123075). Overall, 547 (41%) of differentially expressed lncRNAs were up-regulated in PD and 783 (59%) were down-regulated in PD (Figure 1A). The most differentially expressed lncRNAs in PD versus ID were ENST00000416908 (up; FC 7.3) and T074942 (down; FC 13.5). Only 5.4% (72/1330) of the differentially expressed lncRNAs had a FC ≥ 4.0. Of those, 69 were expressed at a higher level in ID samples while only 3 were expressed at a higher level in the PD samples (Figure 2; Table 2). Using the same Arraystar platform and criteria to analyze mRNA transcripts, 940 of 20,730 mRNAs (4.5%) were differentially expressed with 617 (65.6%) up-regulated in PD versus 323 (34.3%) down-regulated in PD (Figures 1B and 3). The most differentially expressed mRNAs in PD vs ID were PCDHB2 (protocadherin beta 2; up-regulated; FC 4.1) and OR6K2 (olfactory receptor family 6 subfamily K member 2; down-regulated; FC 8.2) (data not shown; NCBI GEO database, accession GSE123075).

**Table 1:** Patient Cohort

	Progressive Disease (PD)^*^ (n=34)	Indolent Disease (ID)^**^ (n=29)
Age at Diagnosis, Minimum (yrs)	42.1	36.2
Age at Diagnosis, Maximum (yrs)	81.4	89.2
Age at Diagnosis, Median (yrs)	54.0	63.4
TTT, Minimum (yrs)	0.1	5.0
TTT, Maximum (yrs)	1.8	10.3
TTT, Median (yrs)	0.45	6.2
Rai 0 at Diagnosis (# of Patients)	13	22
Rai 1 at Diagnosis (# of Patients)	21	7

^*^ TTT ≤ 2 yrs after diagnosis; ^**^ TTT ≥ 5 yrs after diagnosis.

**Table 2:** LncRNAs differentially expressed in PD vs ID Rai 0/I UM CLL patients

Sequence Name^*^	Chr(±)	FC	Relationship	Sequence Name	Chr(±)	FC	Relationship
T074942^1^	chr12(+)	13.5	Intergenic	T089479^**1**^	chr12(+)	5.7	Intergenic
ENST00000435766^2^	chr7(-)	11.0	Intronic Antisense	T004544^**1**^	chr1(+)	5.7	Intergenic
T347014^1^	chr8(-)	10.3	Unknown	ENST00000525429^**2**^	chr11(-)	5.6	Intergenic
ENST00000522339^2^	chr8(+)	9.4	Intergenic	T261919^**1**^	chr4(-)	5.5	Unknown
T183853^1^	chr2(+)	8.9	Intergenic	ENST00000562848^**2**^	chr12(+)	5.5	Sense-Overlapping
NR_110094^3^	chr7(+)	8.7	Intergenic	T315809^**1**^	chr6(-)	5.3	Intergenic
T308148^1^	chr6(-)	8.5	Unknown	T375464^**1**^	chrX(+)	5.2	Unknown
T307709^1^	chr6(-)	8.4	Intergenic	uc002vgl.3^**6**^	chr2(-)	5.1	Intergenic
NR_002947^3^	chr17(+)	8.3	Intergenic	ENST00000446002^**2**^	chr1(-)	5.1	Intergenic
ENST00000453128^2^	chr1(-)	8.2	Intronic Antisense	NR_110545^**3**^	chr21(-)	5.1	Intergenic
T049579^1^	chr10(+)	7.9	Intergenic	T129073^**1**^	chr16(+)	5.1	Intergenic
ENST00000540399^2^	chr12(-)	7.9	Intergenic	T193734^**1**^	chr2(+)	5.0	Intronic Antisense
ENST00000553754^2^	chr14(-)	7.9	Intergenic	T108341^**1**^	chr14(+)	4.7	Unknown
ENST00000449838^2^	chr2(-)	7.7	Intronic Antisense	NR_121599^**3**^	chr9(+)	4.7	Intronic Antisense
ENST00000554679^2^	chr14(-)	7.5	Intronic Antisense	ENST00000552378^**2**^	chr12(+)	4.6	Intergenic
NR_003663^3^	chr2(+)	7.4	Intergenic	ENST00000438380^**2**^	chr9(+)	4.6	Intronic Antisense
TCONS_00026372^4^	chr18(+)	7.3	Intergenic	T076365^**1**^	chr12(+)	4.6	Intergenic
**ENST00000416908^2^**	**chr1(+)**	**7.3**	**Intergenic**	T331223^**1**^	chr7(+)	4.6	Intronic Antisense
T363602^1^	chr9(-)	7.2	Intergenic	ENST00000554412^**2**^	chr15(-)	4.5	Intergenic
ENST00000431401^2^	chr6(-)	7.1	Intergenic	T161929^**1**^	chr18(+)	4.5	Unknown
T096639^1^	chr13(+)	6.8	Intergenic	T343930^**1**^	chr8(+)	4.5	Unknown
TCONS_00006344^4^	chr3(+)	6.7	Intergenic	T084157^**1**^	chr12(-)	4.4	Intergenic
T103502^1^	chr14(-)	6.5	Intergenic	ENST00000607991^**2**^	chr22(+)	4.4	Intronic Antisense
T046734^1^	chr10(-)	6.4	Intergenic	TCONS_00012376^**4**^	chr6(-)	4.4	Intergenic
Uc.432+^5^	chr18(+)	6.4	Intergenic	uc.448-^**5**^	chr19(-)	4.3	Intergenic
ENST00000511222^2^	chr4(+)	6.2	Intronic Antisense	T008095^**1**^	chr1(+)	4.3	Intergenic
T350684^1^	chr8(-)	6.2	Unknown	NR_038345^**3**^	chr7(+)	4.2	Natural Antisense
T248644^1^	chr3(-)	6.1	Unknown	T342775^**1**^	chr8(+)	4.2	Intergenic
ENST00000437897^2^	chr2(+)	6.0	Intronic Antisense	T194929^**1**^	chr2(-)	4.2	Intergenic
T345084^1^	chr8(-)	6.0	Intergenic	T352292^**1**^	chr8(-)	4.1	Unknown
T232736^1^	chr22(-)	5.9	Unknown	ENST00000581648^**2**^	chr18(-)	4.1	Intergenic
uc031qjh.1^6^	chr12(+)	5.9	Natural Antisense	**TCONS_00019584^4^**	**chr11(-)**	**4.0**	**Intergenic**
T305611^1^	chr6(+)	5.9	Sense-Overlapping	T286073^**1**^	chr5(+)	4.0	Unknown
T172505^1^	chr19(-)	5.8	Intergenic	**ENST00000445280^2^**	**chr9(-)**	**4.0**	**Intergenic**
T018494^1^	chr1(-)	5.8	Unknown	ENST00000554458^**2**^	chr14(+)	4.0	Intergenic
T200183^1^	chr2(+)	5.8	Unknown	NR_038923^**3**^	chr11(-)	4.0	Bidirectional

Only those lncRNAs with FC ≥ 4.0 are shown. Chr (±) indicates the genomic location on sense and antisense strands of human chromosomes. FC based on log2 changes. LncRNAs in bold are up-regulated in PD vs ID samples with all other lncRNAs expressed at ≥4 fold higher levels in ID vs PD. ^*^ Indicates the source of the lncRNA sequence name. ^1^ PMID:25599403; ^2^ GENCODE; ^3^ RefSeq (NCBI Reference Sequence Database); ^4^ PMID:21890647; ^5^ PMID:15131266; ^6^ University of California Santa Cruz (UCSC) Known Genes dataset.

**Figure 4:**
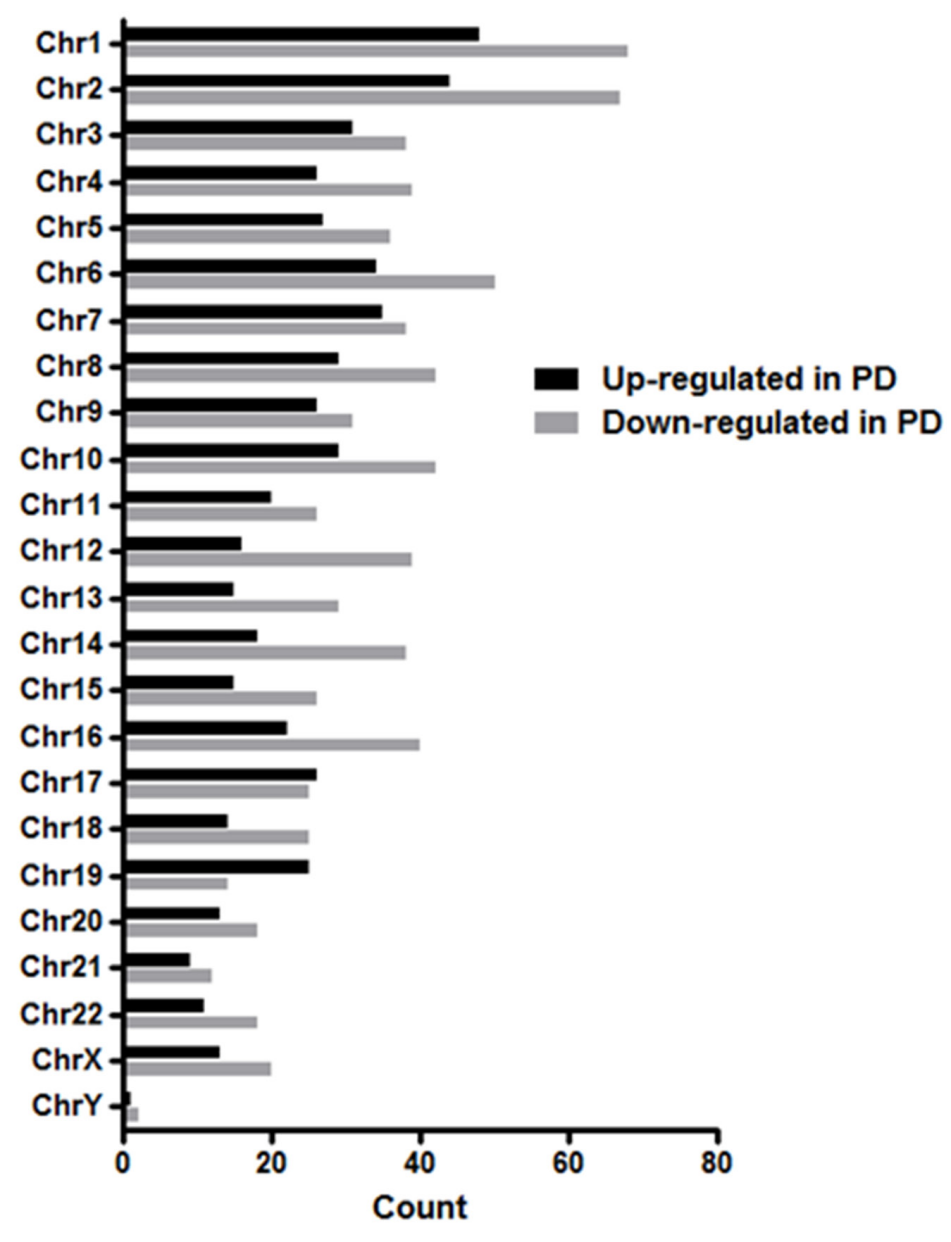
Chromosomal distribution of differentially expressed lncRNAs in PD vs ID early stage CLL The differentially expressed lncRNAs with FC ≥ 2.0 were distributed across all chromosomes.

The differentially expressed lncRNAs were distributed across all chromosomes with 39.5% located on chromosomes 1, 2, 6, 7, 8 and 10 (Figure 4). Given that chromosome 12 occurs as a trisomy in 10-20% of CLL patients and is an indicator of intermediate risk [46], it was conceivable that those patients with trisomy 12 significantly impacted the lncRNA profile with respect to PD or ID CLL. However, when focusing on the trisomy 12 patients only, the differential expression of PD vs ID for those lncRNAs located on chromosome 12 with FC ≥ 2.0 was retained (data not shown) thereby indicating that the extra copy of chromosome 12 in some patients did not underlie differential lncRNA expression.

**Figure 5:**
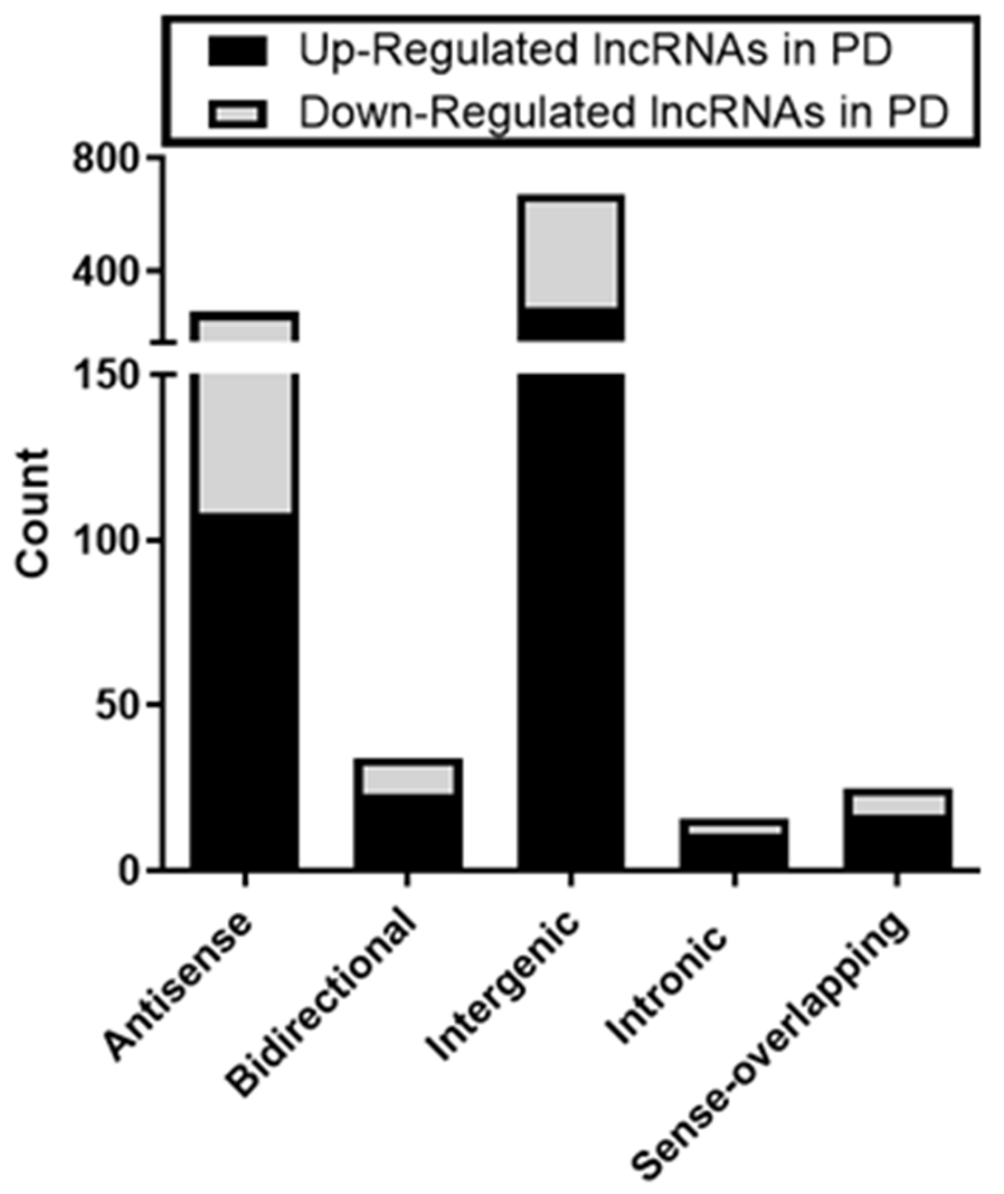
Types of differentially expressed lncRNAs in early stage PD vs ID CLL 1005 of the 1330 lncRNAs could be classified based on relationships to protein coding genes.

LncRNAs can be classified into 5 broad categories based on genomic context and their relationship to protein coding genes [47]. This classification can provide insight into their possible functional roles. Of the 1,330 differentially expressed lncRNAs in this study,1,005 (76%) were classified as antisense, bidirectional, intergenic, intronic, or sense overlapping of introns or exons with 43% being up-regulated in PD and 57% down-regulated in PD across all categories (Figure 5). The majority (67%) of differentially expressed lncRNAs were intergenic (located between annotated coding genes) with 272 intergenic lncRNAs up-regulated in PD and 401 down-regulated. Intergenic lncRNAs are more evolutionarily conserved, can be very tissue-specific and may exhibit active transcription as compared to other types of lncRNAs suggesting functionality [47]. The remaining 33% of classified lncRNAs are oriented in either a sense or antisense direction within exons or introns of protein coding genes and may still carry out a variety of biological roles.

### Gene ontology and pathway analysis

**Figure 6:**
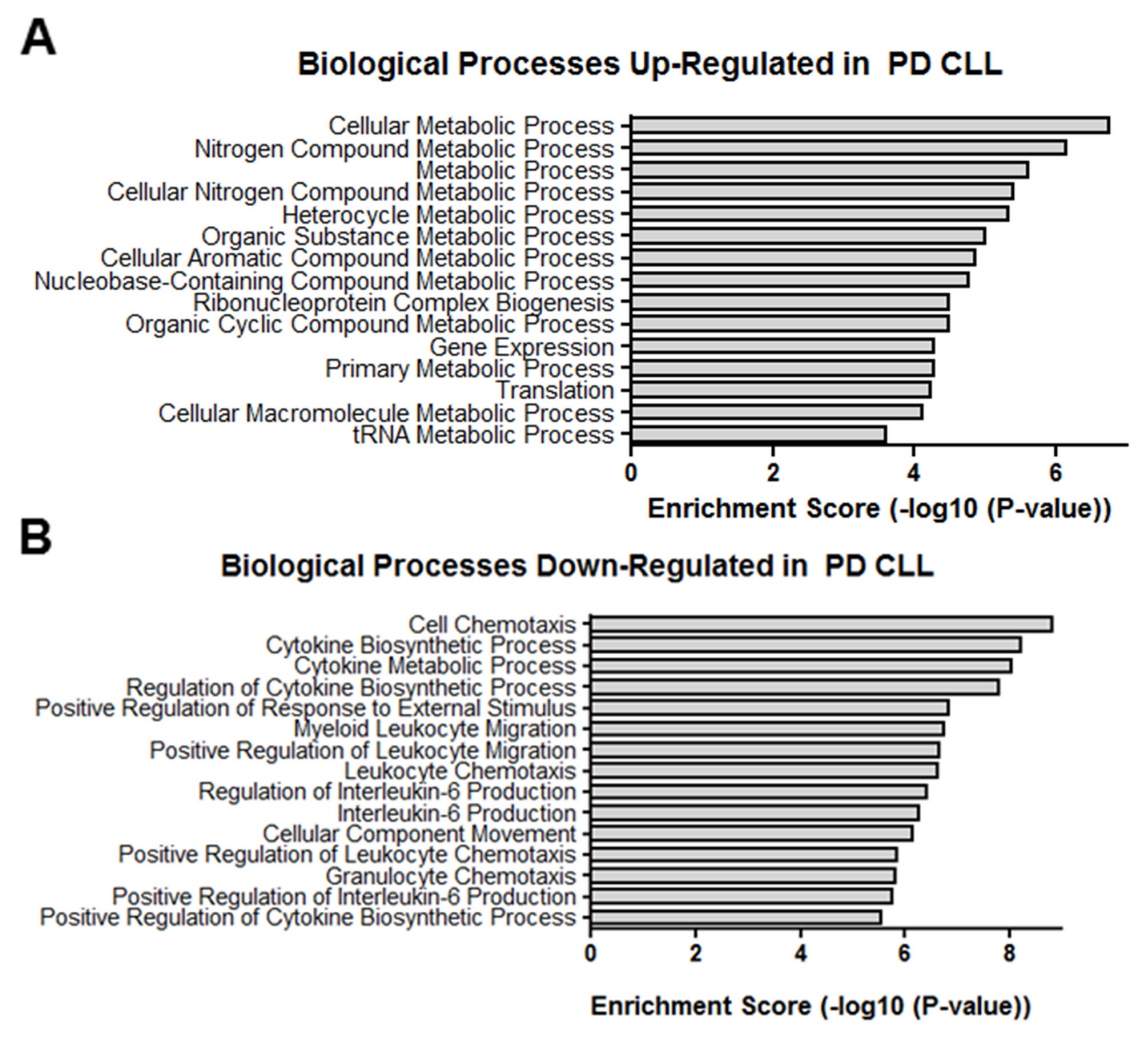
GO biological processes Top GO biological processes enriched in **(A)** up-regulated transcripts and **(B)** down-regulated transcripts differentially expressed in PD vs ID CLL.

**Figure 7:**
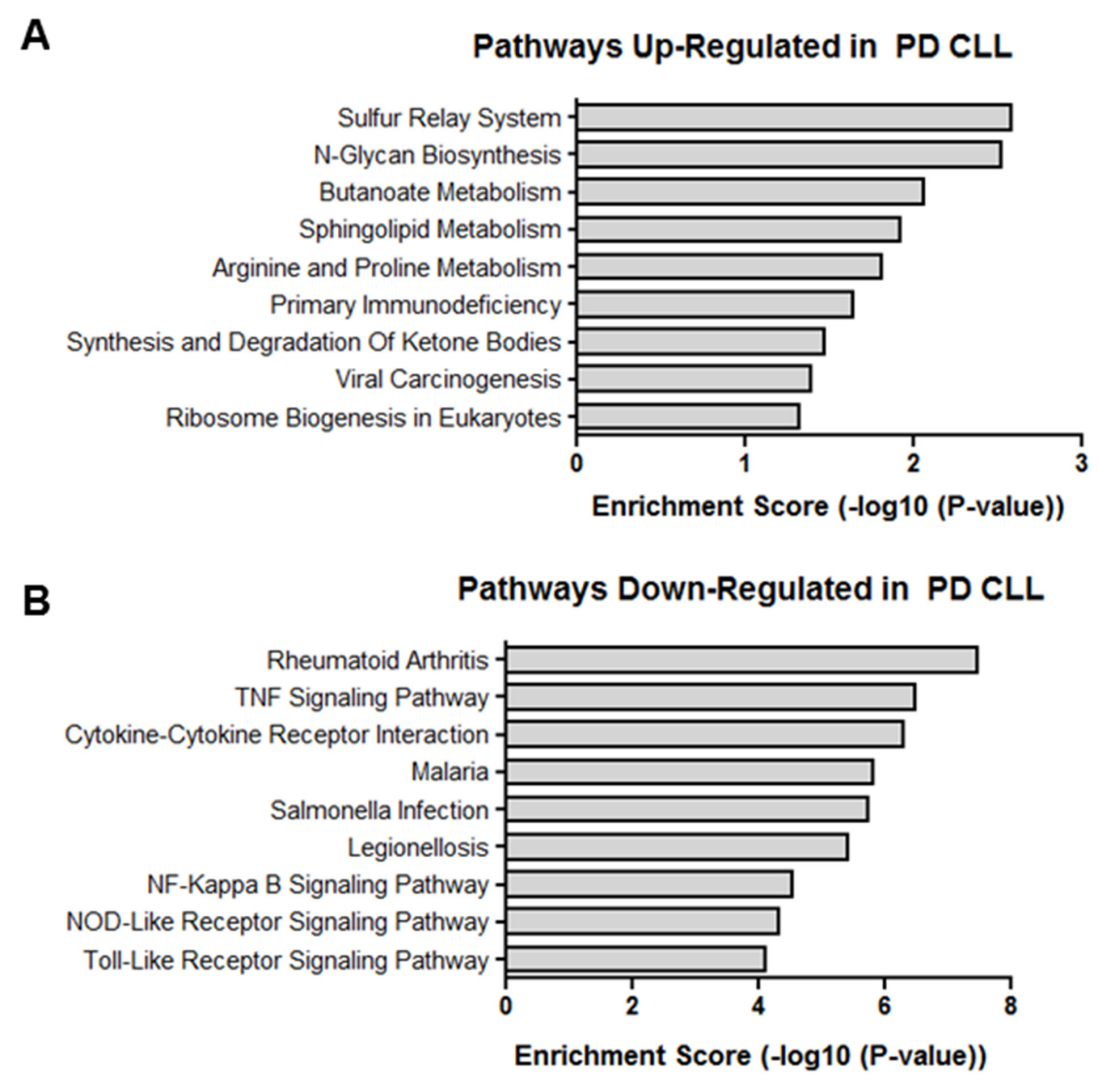
KEGG pathways Enriched pathways in **(A)** up-regulated transcripts and **(B)** down-regulated transcripts differentially expressed in PD vs ID CLL.

Even though the exact target protein coding genes and the biological role of many lncRNAs are unknown, many play a regulatory role in the transcription of protein coding genes [48]. Therefore, the overall function of differentially expressed lncRNAs in PD vs ID samples may be suggested by the mRNA expression profiles of PD vs ID samples. Hence, Gene Ontology (GO) and Kyoto Encyclopedia of Genes and Genomes (KEGG) analysis of the biological processes and pathways associated with differentially expressed mRNAs was done. GO analysis revealed that mRNAs differentially expressed in PD vs ID CLL were linked with over 700 biological processes. The most enriched biological processes associated with up-regulated transcripts in PD samples involved multiple metabolic processes, translation and gene expression while down-regulated transcripts involved chemotaxis and cytokine metabolism and production (Figure 6). KEGG pathway enrichment analysis revealed 49 pathways associated with differentially expressed mRNAs. Pathways associated with up-regulated mRNA in PD were centered again on metabolism but the pathways associated with down-regulated mRNAs featured signaling pathways related to TNF, NF-kappa B, Toll-like receptors, and NOD-like receptors, all of which have been studied in the context of CLL pathophysiology (Figure 7) [49–51].

### Real-time quantitative PCR validation

**Figure 8:**
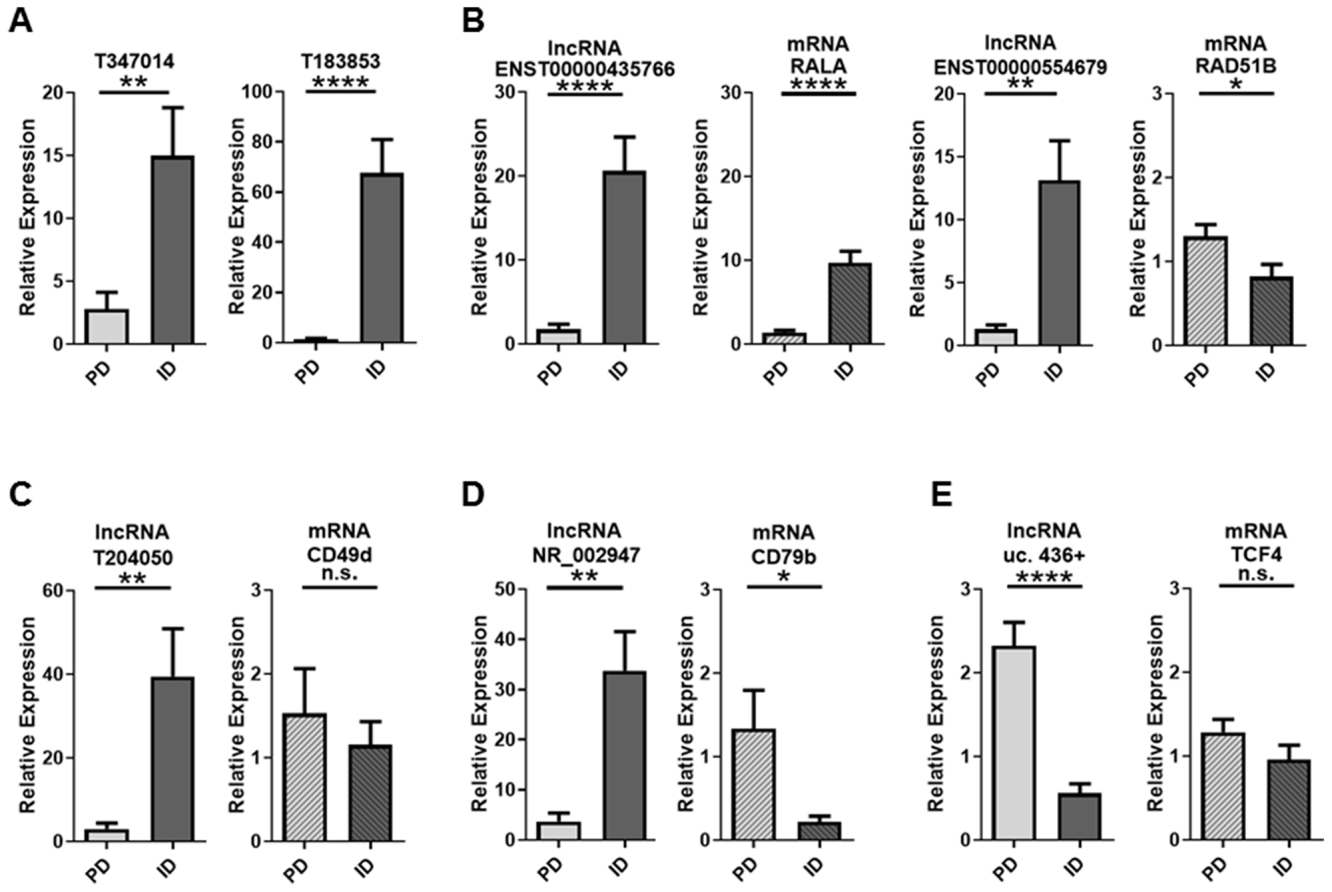
RT-qPCR validation of lncRNAs and associated mRNAs in PD vs ID CLL **(A)** Significant down-regulation by the lncRNAs T347014 and T183853 was confirmed by RT-qPCR in an independent cohort of 10 samples (5 PD and 5 ID). **(B)** ENST00000435766 and ENST00000554679 were also down-regulated by RT-qPCR. Their associated mRNAs were not significantly differentially expressed by microarray analysis but differentially expressed by RT-qPCR. **(C-E)** lncRNAs with known associated mRNAs implicated in CLL had varying regulation patterns. Data are represented as mean +/- SEM; ^*^p<0.05; ^**^p<0.01; ^***^p<0.001; ^****^p<0.0001; n.s.= not statistically significant (two-tailed *t*-test).

Thirty lncRNAs were selected for validation by real-time quantitative PCR (RT-qPCR) based on FC, associated or nearby protein coding genes, and/or relevance to CLL. Protein coding genes are considered associated if the lncRNA is located within 1 kb of the promoter region of the gene(s). LncRNAs beyond 1 kb but within 300 kb are considered intergenic. Using a subset of samples from the original microarray cohort, 25 of the 30 lncRNAs followed the trend of the microarray analysis although only 20 of the 25 reached statistical significance (Supplementary Figure 1). Seven lncRNAs were selected on the basis of either FC or association with protein-coding genes (Table 3; Supplementary Figure 2) and were subjected to further validation using an independent set of 10 CLL patients (5 PD and 5 ID). By microarray analysis, lncRNAs T347014 and T183853 were both down-regulated in PD CLL with FCs of 10.3 and 8.9 respectively. RT-qPCR validated those results with significantly down-regulated expression in PD CLL (Figure 8A). When looking at those differentially expressed lncRNAs with associated protein coding genes, ENST00000435766 (FC=11; associated mRNA RALA) and ENST00000554679 (FC=7.5; associated mRNA RAD51B), were both confirmed to be significantly down-regulated in PD CLL. By RT-qPCR we were able to demonstrate that the associated mRNAs RALA and RAD51B were similarly differentially expressed, despite the lack of differential expression in the original mRNA microarray data (Figure 8B). The lncRNAs T204050 (FC=3.6), NR_002947 (FC=8.3), and uc.436+ (FC=2.8) have known or nearby protein coding genes implicated in CLL biology [52–54]. RT-qPCR expression patterns of all 3 of these lncRNAs were consistent with the microarray data. With respect to the associated mRNAs, only CD79b was differentially expressed (up-regulated in PD) in the validation set (Figure 8C–8E).

**Table 3:** lncRNAs and mRNAs selected for validation by RT-qPCR

lncRNA		Associated or Nearby mRNA	
Sequence Name	FC (Microarray)	Gene Name	FC (Microarray)
T347014	10.3; Down in PD	Not Identified	NA
T183853	8.9; Down in PD	RNF144A	NA
ENST00000435766	11; Down in PD	RALA	NS
ENST00000554679	7.5; Down in PD	RAD51B	NS
T204050	3.6; Down in PD	CD49d	NS
NR_002947	8.3; Down in PD	CD79b	2.7; Up in PD
uc.436+	2.8; Up in PD	TCF4	2.2; Up in PD

Seven lncRNAs and 5 mRNAs of interest were selected for further validation by RT-qPCR. NA=Not assessed by microarray; NS=no statistically significant difference between PD and ID by microarray analysis.

## DISCUSSION

In this study we identified 1,330 lncRNAs and 940 protein coding transcripts that were differentially expressed in a statistically significant manner between PD and ID Rai 0/I UM IGHV CLL using an array based method. We confirmed the differential expression of selected lncRNAs and their associated mRNAs by RT-qPCR including mRNAs that have previously been shown to play a role in CLL. The majority of differentially expressed lncRNAs and mRNAs had a 2-4 FC with only 5.4% of the lncRNAs and 1.1% of mRNAs having a FC ≥ 4.0. Previous studies of lncRNA expression during B cell development show that B cell subsets can be distinguished by their respective lncRNA pattern [34, 36, 55–57]. However, in our study we focused on a very stringent clinical and prognostic marker-defined subset of CLL (Rai stage 0/I UM IGHV) that was distinguished only by TTT and not compared to a normal cell counterpart nor a broader subset of aggressive CLL [45]. Therefore, it is expected that the overall differences in lncRNA expression are relatively subtle; however, our data demonstrate that distinct lncRNA expression patterns within this subset exist.

Of the 30 lncRNAs selected for validation by RT-qPCR, 25 followed the directional trend of the microarray results with 20 reaching statistical significance by both methods. Seven lncRNAs were of particular interest based on high FC (T347014, FC=10.3; T183853, FC=8.9) or known associated or nearby protein coding genes previously linked to cancer (ENST00000435766; ENST00000554679) and specifically CLL (T204050; NR_002947; and uc.436+). The RT-qPCR results of the lncRNAs were consistent with the microarray results. The lncRNAs T347014 and T183853 do not have known associated genes but T183853 does have a nearby coding gene, RNF144A, which has a role in regulating EGF/EGFR signaling and EGF dependent cell proliferation [58] suggesting that further evaluation of this lncRNA may be of value.

The lncRNAs ENST00000435766 and ENST00000554679 have known associated protein coding genes which have been implicated in cancer. RALA (associated with ENST00000435766) is a GTPase and a downstream signaling molecule of RAS whose overexpression has been shown to result in malignant transformation and progression in chronic myelogenous leukemia. This effect can be attenuated by miR-181a, another type of non-coding RNA [59]. In this study, the lncRNA and RALA mRNA were overexpressed in the indolent patient samples, suggesting the lncRNA and associated coding gene may have a different influence on disease pathogenesis in CLL.

RAD51B (associated with ENST00000554679) is a member of the RAD51 family of genes which play a role in homologous recombination and B-cell development [60]. In gastric cancer, RAD51B specifically has been identified as a potential biomarker for poor prognosis [61]. Furthermore, genetic variants in miR-binding sites of RAD51B have been shown to be associated with cervical cancer risk [62]. While germline mutations in RAD51B do not contribute to CLL [63], lack of lncRNA expression may be contributing to RAD51B expression and thus contributing to disease progression.

The lncRNA T204050 was down-regulated in PD by microarray analysis (FC=3.6) and RT-qPCR. Of interest, a nearby coding gene of the T204050 lncRNA is CD49d. We have previously shown that high expression of CD49d on CLL B-cells is a very strong prognostic indicator for aggressive CLL and is associated with shorter TTT [54]. In this scenario, the lncRNA T204050 was more highly expressed in ID CLL, but the associated mRNA for CD49d was not significantly differentially expressed but did follow a directional trend of being up-regulated in PD. However, as noted above, this is a very narrow clinical and prognostically defined subset of CLL and thus a larger cohort will be needed to further delineate if an lncRNA marker is associated with and impacting the expression of the negative prognostic marker CD49d [54].

Biologically relevant in CLL, the lncRNA NR_002947 and mRNA for its associated protein CD79b were found to be differentially expressed by both microarray and RT-qPCR and displayed opposite expression levels suggesting that CD79b expression may be altered by NR_002947. CD79b is a critical component of the B cell receptor and levels of CD79b mRNA have been shown to be higher in UM CLL even though surface expression of CD79b remains low [52]. However, Guo et al have shown that IL-4 restores CD79b protein expression at the post-transcriptional level and speculated that aberrant expression of micro-RNAs may be playing a role [64]. Those findings coupled with our results suggest that lncRNAs may be playing a role in the expression of CD79b and thereby affecting the B-cell receptor in CLL and further studies exploring this link are warranted.

The lncRNA uc.436+ is one of 481 segments longer than 200 base pairs first described by Bejerano et al [65] as being conserved with 100% identity between human, mouse and rat with no insertions or deletions. Calin et al [66] identified some of these regions as being involved in tumorigenesis, particularly human leukemias. While the lncRNA uc.436+ was not identified in Calin’s studies, its nearby coding gene TCF4 is of particular interest in the context of CLL. The TCF/LEF proteins along with their transcriptional cofactor β-catenin are important components of the Wnt signaling pathway. We and others have shown that LEF-1 is aberrantly expressed in CLL B cells acting as a pro-survival factor and a possible marker for aggressive disease [53, 67–69]. Moreover, TCF4 has also been shown to be overexpressed in CLL B cells [70]. Both uc.436+ and TCF4 are significantly overexpressed in PD disease by microarray analysis and the TCF4 gene was found in 12 of the top 15 biological processes identified in our GO analysis of the up-regulated mRNAs in PD but was not identified in the biological processes down-regulated in PD. Furthermore, we validated overexpression of uc.436+ in PD by RT-qPCR in an independent CLL cohort. While not significantly differentially expressed by RT-qPCR, TCF4 did show a directional trend towards being up-regulated in PD supporting further investigation of this lncRNA/mRNA association in PD vs ID CLL.

The IGHV mutation status in CLL has long been used as marker of disease progression with much more unfavorable disease kinetics for CLL clones expressing an UM IGHV. However the precise underlying mechanism for the association with biologically aggressive disease is still unknown. It was postulated at one time that CLL was two distinct diseases arising from B cells at different stages of maturation and therefore could account for the disparities in disease progression [71]. Gene expression profiling, however, found that all cases of CLL have a similar lineage independent of IGHV mutation status and indicated that all CLL B cells are more similar to postgerminal memory B cells [72]. Nonetheless, the great clinical variability in rates of disease progression in Rai 0/I UM IGHV CLL patients suggest undefined biological processes influencing progression. Previous studies have implicated lncRNAs in B cell development and lymphoma cell biology (reviewed in [36]), so it is feasible that lncRNAs may be playing a role in the progression among early stage UM IGHV CLL patients. Indeed, previous studies have found specific lncRNAs that may discriminate prognostic groups [44, 45]. In this study we show for the first time that even within a very specific clinical and prognostic subset of CLL, i.e., Rai 0/I UM IGHV CLL, both well annotated and novel lncRNAs are dysregulated and may be playing a role by altering expression of known associated or nearby genes involved in CLL pathogenesis. Further studies of specific lncRNA functions in CLL are warranted.

## MATERIALS AND METHODS

### Patients and sample collection

All patients provided written informed consent in accordance with the Declaration of Helsinki and the Mayo Clinic Institutional Review Board. The diagnosis of CLL required a B-cell count of ≥ 5 × 10^9^/L along with a CD5+ and κ or λ monoclonal phenotype. For this study the cohort was restricted to Rai stage 0/I patients with UM IGHV (≤ 2.0% deviation from the germline sequence [12, 13]). To evaluate characteristics associated with rapid progression (PD) relative to those who had stable disease (ID) for many years, we identified 34 patients who experienced progression in ≤2 years (TTT ≤2 years) and a separate group who had not experienced progression in ≥5 years (TTT ≥5 years; n=29; Table 1 and Supplementary Table 1).

Peripheral blood from all study subjects was collected within 3 months of diagnosis and prior to any treatment. Mononuclear cells were collected by ficoll density gradient centrifugation (Ficoll-Paque Plus, GE Healthcare, Pittsburgh, PA, USA) and frozen until needed. RNA was isolated using the Trizol method (Life Technologies, Carlsbad, CA, USA) and quantitated on a NanoDrop 2000 spectrophotometer (Thermo Fisher Scientific, Waltham, MA, USA).

### Microarray and computational analysis

RNA was submitted to Arraystar Inc. (Rockville, MD, USA) for lncRNA and mRNA expression profiling and data analysis. The Human lncRNA Array v4.0 covering 40,173 lncRNAs and 20,730 protein coding mRNAs was used. Sample preparation and hybridization were done according to Arraystar standard protocols. Agilent Feature Extraction software (Agilent Technologies, Santa Clara, CA, USA) was used to analyze array images and data analysis was done with GeneSpring GX v12.1 software (Agilent Technologies). After quantile normalization of the raw data, only those lncRNAs and mRNAs with at least 12 Present or Marginal flags were considered for further analysis. Volcano plot filtering identified lncRNAs and mRNAs with statistically significant differences between the 2 groups. Gene Ontology (GO) and Kyoto Encyclopedia of Genes and Genomes (KEGG) analyses were used to identify biological processes and pathway clusters associated with differentially expressed mRNAs. The p-value (≤0.05) as calculated by the Fisher’s exact test denotes the significance of the GO term in the list of differentially expressed mRNAs and the significant enrichment of differentially expressed mRNAs in biological pathways in the KEGG analysis. In both cases the enrichment score is the –log10(p-value). The greater the enrichment score the more significant the pathway correlations. The complete set of lncRNA and mRNA data obtained in this study are available in the NCBI GEO database, accession number GSE123075.

### Real-time quantitative PCR validation

Real-time quantitative PCR (RT-qPCR) was used to confirm the expression of selected lncRNAs and mRNAs in a subset of the microarray samples and in an additional validation set of 10 samples (5 PD and 5 ID). Expression was analyzed using SYBR green reagents (Qiagen, Germantown, MD, USA) on an ABI ViiA 7 Real-Time PCR System (Thermo Fisher Scientific, Waltham, MA, USA). All RT-qPCR experiments were performed in triplicate and included no template controls. Data were analyzed by the 2^δδCt^ method against β-actin for normalization. Primers were designed with Primer-Blast (NCBI) and primer sequences are shown in Supplementary Table 2. Statistical analysis and graphic representation was performed using GraphPad Prism version 5.04 (GraphPad Software, Inc., La Jolla, CA, USA). Results were analyzed with the Student’s t-test.

## SUPPLEMENTARY MATERIALS FIGURES AND TABLES


