# Supplementary material for: Role of long non-coding RNAs in disease progression of early stage unmutated chronic lymphocytic leukemia
Source: Oncotarget. 2019 Jan 1;10(1):60–75. doi: 10.18632/oncotarget.26538 (PMC6343752; doi:10.18632/oncotarget.26538)
Supplement: Supplementary file 1 [file oncotarget-10-60-s001.pdf]

Role of long non-coding RNAs in disease progression of early stage unmutated chronic lymphocytic leukemia

SUPPLEMENTARY MATERIALS

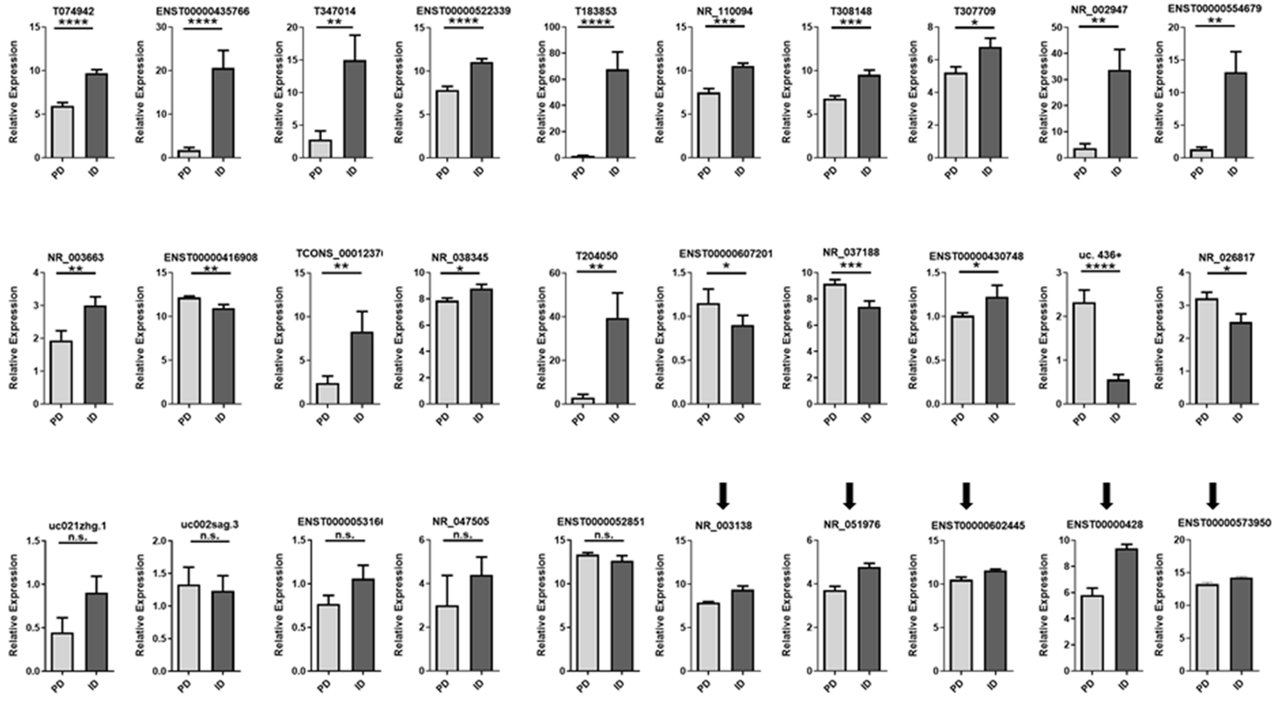

Supplementary Figure 1: RT-qPCR validation of lncRNAs. Thirty lncRNAs were selected for validation by RT-qPCR. Twenty five of the 30 followed the trend of the microarray analysis but only 20 of the 25 reached statistical significance. Arrows indicate those 5 that did not follow the trend of the microarray results. Data are represented as mean +/- SEM; *$p<0.05$; **$p<0.01$; ***$p<0.001$; ****$p<0.0001$; n.s.= not statistically significant (two-tailed *t*-test).

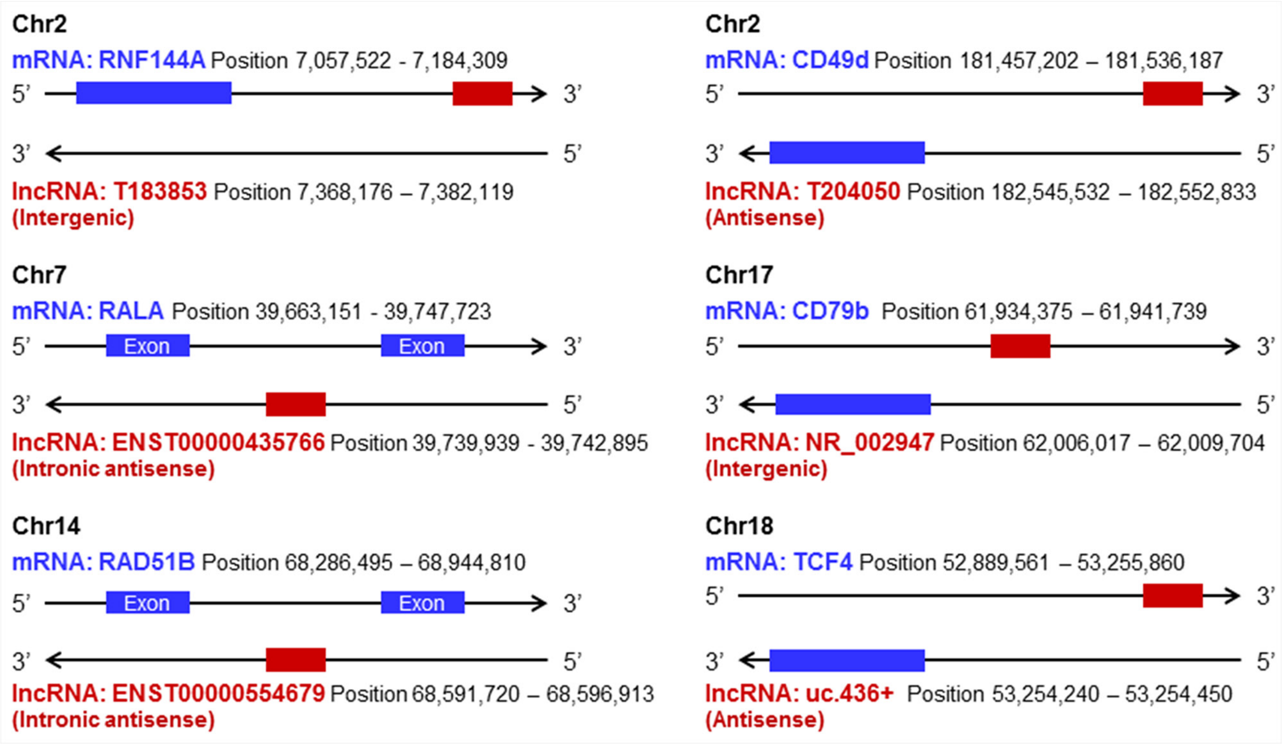

Supplementary Figure 2: Genomic organization of selected lncRNAs and associated proteins. The genomic location and context of the 6 lncRNAs chosen for further validation that have known associated or nearby genes are shown.

Supplementary Table 1: Patient prognostic characteristics

Sample	CD38	CD49d	Zap70	FISH	TK[*]	B2M[***]	Sample	CD38	CD49d	Zap70	FISH	TK	B2M
**PD-1**	−	+	−	Tri 12	+	+	**ID-1**	+	ND	ND	Tri 12	ND	+
**PD-2**	−	+	−	13q-	+	+	**ID-2**	−	−	+	13q-	−	−
**PD-3**	−	ND[**]	ND	11q-	−	−	**ID-3**	+	−	+	Tri 12	+	+
**PD-4**	+	+	+	Tri 12	+	+	**ID-4**	−	ND	ND	Normal	ND	−
**PD-5**	−	+	+	Tri 12	+	+	**ID-5**	+	−	+	11q-	+	+
**PD-6**	+	−	+	13q-	+	+	**ID-6**	+	+	+	Tri 12	+	+
**PD-7**	−	−	+	Normal	+	+	**ID-7**	−	−	−	Normal	+	+
**PD-8**	+	+	+	Tri 12	+	+	**ID-8**	−	ND	ND	13q-	ND	ND
**PD-9**	+	−	+	17p-	+	+	**ID-9**	−	−	−	Normal	+	+
**PD-10**	+	−	+	Normal	+	+	**ID-10**	−	−	+	13q-	+	+
**PD-11**	+	ND	+	Tri 12	+	+	**ID-11**	−	−	−	Normal	+	+
**PD-12**	−	−	+	13q-	+	+	**ID-12**	−	−	−	13q-	−	−
**PD-13**	−	ND	−	11q-	+	+	**ID-13**	−	−	−	11q-	+	+
**PD-14**	+	+	+	Tri 12	+	+	**ID-14**	−	−	−	13q-	+	+
**PD-15**	−	−	+	17p-	ND	+	**ID-15**	+	+	−	Tri 12	+	+
**PD-16**	+	ND	+	13q-	+	−	**ID-16**	−	ND	ND	13q-	ND	−
**PD-17**	−	+	+	13q-	ND	+	**ID-17**	−	−	+	Normal	−	+
**PD-18**	−	+	+	Normal	+	+	**ID-18**	+	+	+	Tri 12	−	+
**PD-19**	−	ND	+	Normal	+	+	**ID-19**	−	−	−	13q-	+	+
**PD-20**	−	+	+	13q-	+	+	**ID-20**	+	+	−	Tri 12	+	−
**PD-21**	+	+	+	11q-	+	+	**ID-21**	+	+	+	Tri 12	+	+
**PD-22**	+	−	+	11q-	+	+	**ID-22**	−	ND	ND	13q-	−	−
**PD-23**	−	−	+	Normal	+	+	**ID-23**	−	+	+	Tri 12	+	+
**PD-24**	−	−	+	Other	+	+	**ID-24**	+	−	+	13q-	+	+
**PD-25**	−	+	+	17p-	+	+	**ID-25**	−	+	−	Tri 12	+	+
**PD-26**	+	−	+	11q-	+	+	**ID-26**	−	−	−	13q-	−	+
**PD-27**	−	−	−	17p-	+	+	**ID-27**	−	−	+	11q-	−	+
**PD-28**	−	−	−	Tri 12	+	+	**ID-28**	+	−	+	Tri 12	+	+
**PD-29**	−	−	+	Normal	ND	+	**ID-29**	−	−	−	Normal	+	+
**PD-30**	+	+	−	11q-	+	+							
**PD-31**	−	ND	+	13q-	+	+							
**PD-32**	−	−	+	11q-	+	ND							
**PD-33**	+	+	+	Normal	+	+							
**PD-34**	−	−	+	13q-	+	+							

[*] TK = Thymidine Kinase; [**] ND = Not Determined; [***] B2M = β2 microglobulin. Positive values are: CD38 > 30%; CD49d > 30%; Zap70 > 20%; TK >10 U/L; B2M > 3.5 mg/L

Supplementary Table 2. Primer sequences for RT-qPCR

Sequence Name (lncRNAs)	Forward primer (5'-3')	Reverse primer (5'-3')
T074942	TGATCTTGGCTCACTGCAAC	ATGCCTGTAATCCCAGCACT
ENST00000435766	CGTATGGTTTCTGCCTGGTT	AGGTTCTGAAGGCTTGTTCG
T347014	GTTCCCGTTAGAGGCTCCTT	CCATTTACATTTGCCGCTTT
ENST00000522339	GAGAAGCCAGCCAGAGTGAC	AGTTCTGTGGTGCTGGGAAC
T183853	GTAGCCTGGGATTTGGGATT	TTCCACCCAAGCTTTTCATC
NR_110094	AGCCAGGTGTGTTTTTCAGG	CATGTGACCCTCCACCTCTT
T308148	AATGTCTTCGTGTGCTTGA	TCAGATTTGTCAGGGCAGTG
T307709	CAACCAGATTCCTGCATTGA	CAGCTCCCTCACAAGACCAT
ENST00000453128	AACCACACTGGAACCTACCG	TTTCCTCCTGTCCACTTTGC
ENST00000554679	ATGAGCCAGCAGAGTCCAGT	CAAGCAAGTGGAAATGCAGA
NR_003663	TTTGCAAAATGGATGGACAA	GGGCAACAGAGAAGAGTTGC
ENST00000416908	GACTTTCAAGTGCCCCAAGA	GCACCTGGGAACAAGTCAT
TCONS_00012376	TTGAGAACAAGCATCCGAGA	GTCCACACTTCCCTCTTCCA
NR_038345	TGTGACAGAGCTCCTTGCTG	GGGAGTCTAGGCCCCTTCTA
T204050	AACCTATCAGCACCGTGGAC	GGAAGGGAAAGAAAGGAGGA
ENST00000607201	GAGCTCTGGAGATTGGTTGC	GGCAAAATAAGGCCCAAGAC
NR_037188	CAAGCCCTGAGTTCTCCAAG	GTTTTCCAGGCTTCAGCATC
ENST00000430748	CCTGAAGTTGTTGTGCCTGA	TCGGGCCAATTCTTCTATTG
Uc.436+	AAGAGAAGGTCCTGGGGAAA	AAGCTGACCCAAGGAAATGA
NR_026817	CCCCCGATAATCATCACATC	AGCCAAGCTTGAGGACAAAA
uc021zhg.1	CTTAGCCTAGCCTGCCTTCA	ACCGTGGTGTGAAAGTGACA
uc002sag.3	ACTGCACTTGAAGGCTTGGT	CAAAAGGGTCCGCAAAATAA
ENST00000531661	ACAGTGCAGTGGTGGTTCAA	ATTTGCTTCAAATGCCGAAC
NR_047505	ACAACTGCATGTGGCCTGTA	TCAGTCTGGGCTCCAAAGTC
ENST00000528510	TGTCCAGCTTCTGGGTTAGG	CGGATTCCCTTATGGAGTGA
NR_003138	TCTGAGTTTCCAGCCTCGTT	ATTTCTCACGATGGGTCCAA
NR_051976	GGCCGATTTGAGAGAGTGAG	CAAGGATTGTTGGGAAGGTC
ENST00000602445	TGGCATGTTACTGAGCCTTG	TGTGAAGGTTGTTGCAGAGC
ENST00000428188	GGTGGTACAGGCTCAACTGG	GCCTCAACAAGGTGAGGAGA
ENST00000573950	GGCAGCTTCACAACAGGTTT	CGTGTGACTGTGAGCAAGGT
**Gene Name (mRNAs)**	**Forward primer (5'-3')**	**Reverse primer (5'-3')**
RALA	GAGACACAAAATGGCTGCAA	GGACTTCCTCCCCATCTAGC
RAD51B	GAGCGCTGGAACGTTTATTC	TTCAAGGCAGGCAAGCTTAT
CD49d	AGTTCCTCCCCCTGTCAGAT	CCTTTTTGGGACCAGTTTCA
CD79b	GTCATGGGATTCAGCACCTT	CATAGGTGGCTGTCTGGTCA
TCF4	TTATGAATCGCAGACGCAAG	GCACAAATGCATTGTTCCAC
β-actin	Qiagen RT^2 qPCR Primer Assay Cat# 33001 PPH00073E	
